# Prevalence and Severity of Hypermetropia and Astigmatism Among Druze Children From the Golan Heights

**DOI:** 10.1155/joph/9259218

**Published:** 2026-04-30

**Authors:** Avi Schwalb, Aviv Vidan, Michael Yulish, Yuval Cohen, Otzem Chassid

**Affiliations:** ^1^ Department of Ophthalmology, Hillel Yaffe Medical Center, Hadera, Israel, hy.health.gov.il; ^2^ Azrieli Faculty of Medicine, Bar-Ilan University, Safed, Israel, biu.ac.il; ^3^ Department of Ophthalmology, Ziv Medical Center, Safed, Israel, ziv.org.il

**Keywords:** astigmatism, Druze, epidemiology, hypermetropia, pediatric ophthalmology, refractive errors

## Abstract

**Background:**

Refractive errors in childhood, if untreated, may lead to long‐term visual and developmental complications. Studies have shown differences in the prevalence and severity of refractive errors among various ethnic groups.

**Purpose:**

To evaluate the prevalence and severity of hypermetropia and astigmatism among Druze children from the Golan Heights compared to the general population in northern Israel.

**Methods:**

A retrospective clinic‐based analysis of 1116 children aged 1–12 years, examined at the Pediatric Ophthalmology Clinic at Ziv Medical Center between 2016 and 2018. Subjects were divided into groups based on their place of residence. Prevalence of hypermetropia and astigmatism was compared between groups using chi‐square tests, and severity was compared using the Mann–Whitney *U* test.

**Results:**

Hypermetropia and astigmatism were significantly more prevalent in Druze children than in non‐Druze children (60.9%, 95% CI: 54.6–66.8 vs. 48.1%, 95% CI: 44.8–51.4, *p* < 0.01 for hypermetropia; 58.8%, 95% CI: 52.6–64.9 vs. 46.8%, 95% CI: 43.5–50.1, *p* = 0.01 for astigmatism). Druze children also exhibited higher severity of both hypermetropia (*p* = 0.017) and astigmatism (*p* = 0.046) compared to non‐Druze children.

**Conclusion:**

Hypermetropia and astigmatism are more prevalent and severe in Druze children of the Golan Heights compared to the general population of northern Israel. Screening tests may be advised for this population to enable early diagnosis and treatment, thereby preventing visual and developmental complications.

## 1. Introduction

Refractive errors in childhood, if untreated, may have long‐term visual and developmental complications. These complications can be prevented by early diagnosis and treatment with appropriate optical interventions, such as glasses or contact lenses [1, 2]. The impact of uncorrected refractive errors extends beyond vision, affecting cognitive development, educational outcomes, and quality of life [3].

During early childhood, particularly in preschool years, adequate vision is crucial for the acquisition of critical skills, including reading and writing. Studies have demonstrated that untreated hypermetropia negatively affects early literacy. For example, a U.S. study using the Test of Preschool Early Literacy (TOPEL) found that children with untreated hypermetropia scored significantly lower than those with emmetropia [1].

Amblyopia, a leading cause of visual impairment in children, may develop when refractive errors remain uncorrected during critical periods of visual development. Although treatment has traditionally been considered most effective before 9–10 years of age, recent evidence suggests that some neural plasticity may persist beyond this period [4–6].

Numerous studies have been conducted to characterize the prevalence of refractive errors in children. They have shown an inverse association between age and the prevalence of hypermetropia [7], as well as differences in the prevalence and severity of hypermetropia and astigmatism among various ethnic groups in the United States [8–10]. A recent meta‐analysis found that childhood hypermetropia prevalence is relatively higher in the Eastern Mediterranean Region compared to other regions [11]. These ethnic and regional variations suggest a complex interplay between genetic and environmental factors in the development of refractive errors [12].

In a study conducted in Israel by Ore et al. in 2014, 1708 Jewish and Arab children from 70 schools in the north of the country were examined. The results showed that the prevalence of hypermetropia was 3.28 times higher in children aged 6‐7, with prevalence 1.85 times higher among Arab children compared to Jewish children [13, 14]. These findings highlight the importance of considering ethnic and cultural factors in pediatric vision screening programs.

While global and regional studies demonstrate substantial variation in refractive error prevalence between populations, and Israeli studies have reported differences between Jewish and Arab children, data on small and relatively isolated ethnic groups, particularly the Druze population of the Golan Heights, remain limited.

The Druze community, characterized by a relative genetic isolation, high rates of consanguinity, and unique cultural practices, presents an interesting population for studying refractive errors [15]. Limited access to pediatric ophthalmology services in the Golan Heights may further influence both the development and detection of refractive errors.

Over the past decade, our clinical impressions at the pediatric ophthalmology clinic at Ziv Medical Center suggested a relatively high burden of significant refractive errors among Druze children from the Golan Heights. These unpublished observations motivated the present structured analysis.

There is limited published research focusing specifically on the Druze population. Based on clinical observations, we aimed to compare the prevalence and severity of hypermetropia and astigmatism among Druze children aged 1–12 years from the Golan Heights with those of other children from northern Israel attending the same tertiary ophthalmology clinic. Although this clinic‐based study cannot determine the true population prevalence, such comparisons provide important insight into ethnic disparities and may help guide future public health planning.

## 2. Materials and Methods

This study is a retrospective analytical study, approved by the institutional ethics committee, adhering to the Declaration of Helsinki. Data were collected from patient files of children aged 1–12 who were examined at the Pediatric Ophthalmology Clinic between 2016 and 2018. Cycloplegia was induced using cyclopentolate 1% and tropicamide. Refractions were performed by a pediatric ophthalmologist. All children aged 1–12 who underwent a cycloplegic refraction test within this time period were included in the study; no records were excluded. Collected data included age, sex, place of residence, and refractive errors.

Patients were categorized based on their place of residence: Druze children from the Golan Heights and non‐Druze children from other northern Israeli localities. Non‐Druze children were further divided into Jewish and Arab subgroups (patients from localities with predominantly Jewish or Arab populations, according to the 2022 census data by the Israeli Central Bureau of Statistics [CBS]).

Refractive errors were categorized as hypermetropia or astigmatism, with severity classified as mild, moderate, or severe, according to the classification system used by Ore et al. [13, 14]. Hypermetropia was defined as mild (+1.00 to +2.75 D), moderate (+3.00 to +4.75 D), and severe (≥ +5.00 D), while astigmatism was defined as mild (0.75–2.00 D), moderate (2.25–3.75 D), and severe (≥ 4.00 D), as shown in Table 1. Myopia was defined as a cycloplegic spherical equivalent of ≤ −0.5 D. This classification allows for a standardized comparison with previous studies in the region.

**TABLE 1:** Classification of refractive error severity according to Ore et al. [13].

	Hypermetropia	Astigmatism
Mild	+1.00 to +2.75 D	0.75 to 2 D
Moderate	+3.00 to +4.75 D	2.25 to 3.75 D
Severe	≥ +5.00 D	≥ +4.00 D

For prevalence calculations, one eye with a refractive error was sufficient to classify a patient into a refractive error group. For severity analysis, both eyes were evaluated, as the degree of refractive error can differ between eyes in the same child. Analyzing both eyes allows a more complete description of the range and distribution of refractive error severity within this population. This approach aligns with established methodologies in pediatric ophthalmology research [16].

Statistical analyses were conducted using SPSS (IBM Corp., version 27) and G∗Power3.1.7 software. A priori power analysis was conducted for a chi‐square test using an expected medium effect size (*w* = 0.3), alpha = 0.05, power = 0.90, and an allocation ratio of approximately 1:3.6, confirming that the available sample size (*n* = 1116) exceeded the minimum required for detecting between‐group differences. Prevalence was compared between groups using chi‐square tests. Severity, classified as mild, moderate, or severe, was compared between groups using the Mann–Whitney *U* test, an appropriate nonparametric test for ordinal data. Severity distributions across categories were also compared between groups using chi‐square tests. Odds ratios were derived from cross‐tubulations. A *p* value of < 0.05 was considered statistically significant.

This study was approved by the Institutional Ethics Committee of Ziv Medical Center (Approval No. 0003–18‐ZIV), and conducted in accordance with the principles of the Declaration of Helsinki. The requirement for informed consent was waived due to the retrospective design. All data were anonymized to ensure patient confidentiality.

This study adhered to the STROBE guidelines for observational research. A completed STROBE checklist is provided in the Supporting Information.

## 3. Results

**FIGURE 1:**
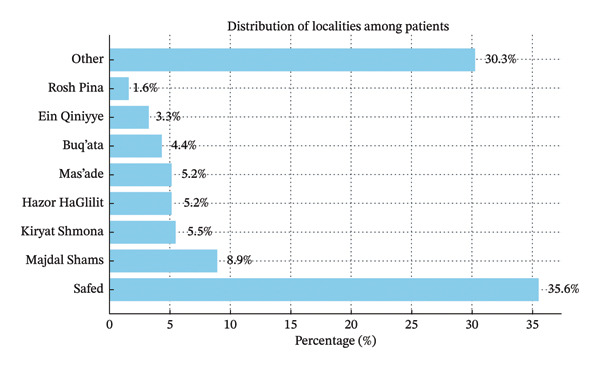
Distribution of localities among the patients included in the study.

Data were available for 1116 patients examined between 2016 and 2018, of which 243 (21.8%) were Druze children from the Golan Heights, and 873 (78.2%) were non‐Druze children, representing the general population in northern Israel. In addition, to compare different populations, two subgroups were created: children from only‐Jewish localities (534 children) and children from only‐Arab localities (76 children). Figure 1 presents the distribution of referral localities, showing that most patients came from Safed (35.6%) and Druze towns of the Golan Heights (21.8%). This reflects both the hospital’s catchment area and potential referral bias related to geographic accessibility.

The average age of subjects in the Druze and non‐Druze groups was identical (mean: 6.2 years; median: 6 years). No significant gender differences were observed (51.4% males, 48.6% females).

The prevalence of hypermetropia among Druze children from the Golan Heights was significantly higher than that among non‐Druze children (60.9%, 95% CI: 54.6–66.8 vs. 48.1%, 95% CI: 44.8–51.4, *p* < 0.01). Similarly, the prevalence of astigmatism was higher among Druze children (58.8%, 95% CI: 52.6–64.9) compared with non‐Druze children (46.8%, 95% CI: 43.5–50.1; *p* = 0.01). These findings are consistent with studies in other ethnically distinct populations that have shown variations in refractive errors prevalence [17, 18].

Comparing the Druze group with the Jewish subgroup showed pronounced differences. The prevalence of hypermetropia and astigmatism among the Jewish children was 46.4% (95% CI: 42.3–50.7) and 43% (95% CI: 38.8–47.2), respectively, compared to 60.9% and 58.8% in the Druze children, (*p* < 0.01).

The prevalence of hypermetropia among the Druze was also higher than among the Arab subgroup (60.9% vs. 47.4%, 95% CI: 36.5–58.4; *p* = 0.037). No significant difference was observed between these groups regarding the prevalence of astigmatism (58.8% in Druze children compared to 60.5%, 95% CI: 49.3–70.8 among Arabs).

**FIGURE 2:**
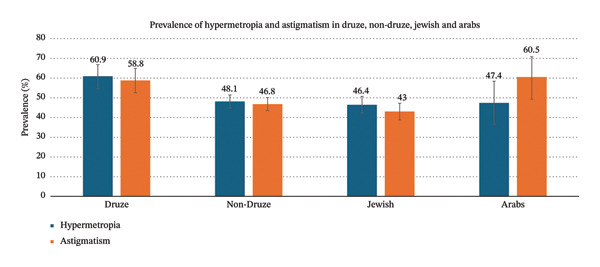
Comparison of the prevalence of hypermetropia and astigmatism between Druze, non‐Druze, Jewish, and Arab groups.

Figure 2 shows a comparison of the prevalence of hypermetropia and astigmatism between the Druze group and the non‐Druze, including the subgroups of Jewish and Arab populations.

**FIGURE 3:**
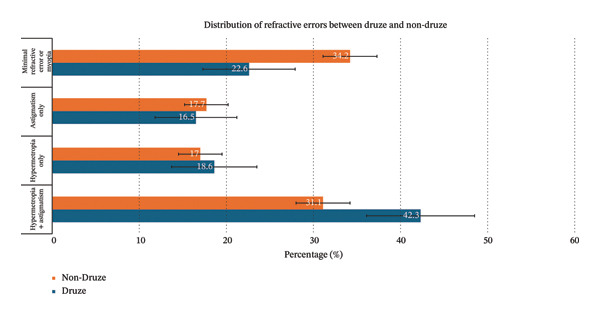
Distribution of refractive errors between Druze and non‐Druze.

Given the overlap between hypermetropia and astigmatism, we assessed how many children presented with neither condition (children with neither hypermetropia nor astigmatism were classified as having minimal refractive error or myopia). Figure 3 illustrated the comparison between the Druze and the non‐Druze populations. Among the non‐Druze children, 34.2% had neither hypermetropia nor astigmatism, whereas only 22.6% of Druze children fell into this category (*p* = 0.01). Furthermore, the odds ratio of having hypermetropia or astigmatism was 1.78 times higher (95% CI: 1.27–2.48) in the Druze children of the Golan Heights compared to the general population of northern Israel. These findings suggest that genetic and/or environmental factors specific to the Druze population may be associated to the higher prevalence of refractive errors [19].

Regarding myopia, 162 children (14.5%) were classified as myopic (cycloplegic spherical equivalent of ≤ −0.5 D). Among Druze children, 26 of 243 (10.7%, 95% CI 6.8%–14.6%) had myopia, compared with 136 of 873 (15.6%, 95% CI 13.3%–17.9%) in the non‐Druze group (*p* = 0.04).

Severity analysis revealed that overall severity was significantly higher for both hypermetropia (*p* = 0.017) and astigmatism (*p* = 0.046) among the Druze children compared to non‐Druze children. Specifically, 27.7% of Druze children with hypermetropia were classified as having severe hypermetropia, compared to 19.3% of non‐Druze children. Similarly, 31.2% of Druze children with astigmatism had moderate to severe astigmatism, compared to 24.4% of non‐Druze children. Table 2 summarizes the differences in severity of hypermetropia and astigmatism between Druze and non‐Druze children. The higher severity of refractive errors in Druze children underscores the importance of early vision screening in this population [20].

**TABLE 2:** Distribution of refractive error severity among Druze and non‐Druze children with hypermetropia and/or astigmatism.

Condition	Severity	Druze (%, *n*/*N*)	Non‐Druze (%, *n*/*N*)	*p* value	Odds ratio (95% CI)
Hypermetropia	Mild	54.1% (80/148)	62.9% (264/420)	0.015	0.7 (0.52–0.93)
	Moderate	18.2% (27/148)	17.9% (75/420)	0.98	1.02 (0.71–1.48)
	Severe	27.7% (41/148)	19.3% (81/420)	0.006	1.6 (1.15–2.22)

Astigmatism	Mild	68.8% (98/143)	75.6% (309/409)	0.039	0.71 (0.52–0.97)
	Moderate	24.3% (35/143)	20.2% (83/409)	0.19	1.27 (0.91–1.78)
	Severe	6.9% (10/143)	4.2% (17/409)	0.11	1.69 (0.93–3.07)

*Note:* Overall severity comparisons were performed using the Mann–Whitney *U* test. Category‐specific comparisons were performed using chi‐square tests. Odds ratios are presented with the non‐Druze as the reference (OR > 1 = higher odds among Druze).

## 4. Discussion

This study demonstrated that hypermetropia and astigmatism are both more prevalent and severe in Druze children from the Golan Heights compared to their non‐Druze peers in northern Israel (prevalence of 60.9% vs. 48.1%, *p* < 0.01 for hypermetropia; 58.8% vs. 48.8%, *p* = 0.01 for astigmatism). The higher severity of refractive errors observed in Druze children is particularly concerning, as untreated hypermetropia and astigmatism are well‐established risk factors for amblyopia and strabismus, which can lead to permanent visual impairment if not treated during the critical period of visual development [21–23]. Moreover, children with significant refractive errors are also at risk of lower educational performance and educational disadvantage that may persist into adulthood [24].

The higher prevalence and severity of refractive errors in Druze children raise several important considerations. Genetic factors may be associated with the higher prevalence observed, given the relative genetic isolation and high rates of consanguinity in the Druze community, which may increase the expression of recessive or polygenic traits related to ocular development, as suggested by familial and twin studies demonstrating a substantial heritable component in refractive errors. [25, 26]. Environmental factors, such as lifestyle, diet, and exposure to outdoor light, which have been implicated in refractive error development, should also be investigated [27, 28].

The findings of this study also highlight the importance of culturally sensitive approaches to pediatric vision care. The Druze community, with its unique cultural practices and beliefs, may have specific barriers to accessing eye care services or adhering to prescribed treatments [29]. Understanding and addressing these barriers is crucial for implementing effective vision screening and treatment programs.

The prevalence of hypermetropia observed in our cohort (60.9% in the Druze group and 48.1% in the non‐Druze group) is considerably higher than the pooled estimate reported for the Eastern Mediterranean Region (approximately 6.33%) in the recent meta‐analysis by Alrasheed and Challa [11]. However, as our study was based on a clinic‐attending population, these figures likely overestimate the true prevalence due to referral bias.

Because of the nature of this study, which examined children who are already under ophthalmologic care, the exact prevalence of these refractive errors in the Druze population remains unclear. However, by comparing the Druze children with other population groups, we can conclude that their prevalence of hypermetropia and astigmatism is among the highest in the region. This observation aligns with studies in other ethnically distinct populations that have shown variations in refractive error prevalence [30, 31].

Analyzing the distribution of localities from which our subjects were from, it is evident that the Ziv Medical Center in Safed primarily serves the Safed subdistrict and the northern Golan Heights. Our study sample reflects an over‐representation of residents from Safed (35.5%) and the Druze councils from the Golan Heights (21.8%) when compared to their respective population proportions of 22% and 15%, based on CBS census data from 2022. For example, Kiryat Shmona, which has a population comparable to that of the four Druze localities of the Golan Heights together, only represented 5.5% of our study sample.

The over‐representation of Safed residents can be explained by their proximity to the ophthalmology clinic at Ziv Medical Center. In contrast, the higher representation of Druze children could be attributed to two possible factors: a lack of access to local ophthalmology services in the Golan Heights, leading to delayed diagnosis and treatment, and a potentially true increased prevalence of refractive errors among the Druze population itself. This interpretation remains speculative, however, and should be confirmed in future population‐based or qualitative studies examining healthcare utilization patterns.

The disparity in healthcare access raises important public health considerations. Geopolitical and socioeconomic conditions in the Golan Heights may further limit access to preventive pediatric vision care and may contribute to delayed diagnosis and treatment of refractive errors in Druze children. This situation calls for targeted interventions, such as mobile eye clinics or telemedicine initiatives, to improve access to eye‐care services in underserved areas [32].

This study has several limitations that should be considered when interpreting the findings. First, its retrospective design limits control over potential confounding variables and introduces the possibility of selection bias. Second, as the study population comprised children attending a tertiary ophthalmology clinic, referral bias must be considered as children with suspected strabismus or visual complaints are more likely to be referred, which may inflate the proportion of hypermetropic patients in a clinic‐based sample. Moreover, data on potential confounding factors such as socioeconomic status, refractive history of parents or siblings, cultural differences in health‐seeking behavior, and environmental exposures, including screen time and outdoor activity, were not available and may have influenced refractive outcomes.

Severity analyses used eye‐level data without clustering adjustment, which may slightly overestimate statistical significance. Formal interobserver standardization of cycloplegic refraction was not formally assessed. In addition, we did not perform age or sex‐adjusted statistical analyses; however, the two groups had similar age and sex distributions, and this limitation is acknowledged.

Another limitation relates to the classification of ethnicity. In this study, ethnicity was inferred from place of residence. According to the 2022 Israeli CBS census data, Druze towns in the Golan Heights (Majdal Shams, Buq’ata, Mas’ade, and Ein Qiniyye) are 99.6%–100% Druze, making residence a highly reliable proxy for Druze ethnicity. A similar principle was applied to define the Jewish‐only group (patients from localities with over 90% Jewish population, such as Safed, Kirya Shmona, Hatzor HaGlilit, and Rosh Pina) and the Arab‐only group (patients from localities with over 95% Arab population in the Upper Galilee region, such as Tuba‐Zangariya, Jish, Ghajar, and Nahf, among others). Moreover, in Israel, personal and family names generally correspond with religious or ethnic affiliation, further supporting classification accuracy. Nonetheless, rare misclassification is possible, especially in mixed‐ethnicity households, and results should be interpreted with this in mind.

An additional limitation relates to the time period of data collection. The study included children examined between 2016 and 2018, prior to the COVID‐19 pandemic. Multiple international studies have demonstrated a shift in refractive error patterns following the pandemic, generally toward increased myopia and reduced hypermetropia, likely due to changes in visual environment such as increased near work and reduced outdoor activity [33]. The effect of these environmental changes on the Druze pediatric population is currently unknown, and the present data may therefore not fully represent the current refractive profile of this community. Future studies are needed to determine whether similar trends have occurred in this population.

Considering the developmental risks posed by untreated refractive errors and the limited access to pediatric ophthalmology services, early screening and intervention programs are crucial for the Druze population of the Golan Heights. The results suggest that the Druze population should be considered at higher risk for refractive errors, warranting targeted screening programs in schools and preschools to ensure early diagnosis and treatment. Such programs have been shown to be effective in other high‐risk populations [34, 35].

The implementation of these screening programs should be accompanied by public health education initiatives to raise awareness about the importance of early vision care among Druze communities. Engagement with community leaders and integration of vision screening into existing health and educational systems, as well as the establishment of periodic mobile eye clinics, could improve uptake and effectiveness of these interventions [36]. Although genetic factors may contribute to the high prevalence observed, these findings should primarily be viewed as a health‐equity signal, underscoring the need for prioritized screening and improved access to care. Future genetic studies may help clarify underlying hereditary influences associated with refractive errors [26]. Prospective population‐based epidemiological studies, together with assessment of environmental exposures and longitudinal evaluation of refractive error progression, are recommended to accurately quantify the true prevalence of refractive errors in the Druze population and to better inform future planning of regional eye‐care resources.

## 5. Conclusions

This study demonstrates a higher prevalence and severity of hypermetropia and astigmatism among Druze children from the Golan Heights compared to the general population in northern Israel. These findings highlight the need for targeted screening programs and early intervention in this population to prevent potential visual and developmental complications associated with untreated refractive errors.

The results underscore the importance of considering ethnic and cultural factors in pediatric vision care and suggest the need for further research into the genetic and environmental factors contributing to refractive errors in the Druze population. Implementation of culturally sensitive community‐based vision screening programs could significantly improve visual outcomes and overall development in this at‐risk population.

Further studies are needed to better understand the exact prevalence of refractive errors in Druze children, to investigate the underlying causes of the observed disparities, and to evaluate the effectiveness of early screening programs in this population. Long‐term follow‐up studies would also be valuable to assess the impact of early detection and treatment on visual, educational, and quality of life outcomes in this unique population.

## Funding

The authors declare that no funds, grants, or other support were received during the preparation of this manuscript.

## Conflicts of Interest

The authors declare no conflicts of interest.

## Data Availability

The data used to support the findings of this study are available from the corresponding author upon request.
